# The Effect of Health Insurance Status on School Attendance

**DOI:** 10.7759/cureus.35366

**Published:** 2023-02-23

**Authors:** Nitya Ramalingam, Evanelys Darias, Emily Soeder, Grettel Castro, Juan Lozano

**Affiliations:** 1 Pediatrics, Florida International University, Herbert Wertheim College of Medicine, Miami, USA; 2 Emergency Medicine, Florida International University, Herbert Wertheim College of Medicine, Miami, USA; 3 Diagnostic Radiology, Florida International University, Herbert Wertheim College of Medicine, Miami, USA; 4 Medical and Population Health Sciences Research, Florida International University, Herbert Wertheim College of Medicine, Miami, USA; 5 Translational Medicine, Florida International University, Herbert Wertheim College of Medicine, Miami, USA

**Keywords:** insurance coverage, school attendance, adverse childhood events, school absenteeism, lack of health insurance

## Abstract

Introduction

Educational achievement is impacted by a student’s ability to be present and motivated in the classroom. Since health and education influence one another, disparities in health insurance status among children may exert educationally relevant consequences. However, the association between health insurance coverage and school absenteeism remains poorly understood. Our study aims to assess the association between not having/having gaps in health insurance coverage and an increased number of missed school days.

Methods

A historical cohort study was performed via secondary analysis of data collected as part of the 2018 National Survey of Children’s Health (NSCH). We included children enrolled in school between the ages of 6-17 years and who provided answers to survey questions involving our two variables of interest: health insurance status and missed school days. Our data analysis included 1) a descriptive analysis of the baseline sample characteristics, 2) a bivariate analysis to determine the association between baseline characteristics/confounding variables and the outcome, and 3) a multivariable regression analysis using logistic regression to determine the association of interest while controlling for potential confounding variables.

Results

A total of 21,498 respondents were included. The unadjusted odds of chronic absenteeism were found to be 16% (OR=1.16) higher in children without insurance or with gaps in insurance compared to children with consistent insurance throughout the year, but the association was not statistically significant (95% CI 0.74 - 1.82, p=0.051). After adjustment by age, sex, race, Hispanic ethnicity, and confounding variables, the odds of chronic absenteeism in children without insurance or with gaps in insurance remained statistically insignificant (aOR=1.05; 95% CI 0.64 - 1.73, p=0.848) compared to those with consistent insurance coverage.

Conclusions

According to our analysis, the data do not support our hypothesis of a significant difference in missed school days (greater than or equal to 11 missed days of school) among those children who had health insurance compared to those without health insurance/had gaps in insurance coverage.

## Introduction

This article was previously presented as a poster at the 2022 American Public Health Association Annual Meeting on November 7th, 2022.

School absence may be one of the many factors impacting school performance in children [1]. It has been estimated that poor attendance correlates with poorer grades and hinders academic performance [2]. There is evidence showing an association between children of low-income backgrounds and school attendance [3]. Prior studies have also analyzed the association of unaffordable dental care and correlated it with increased school absences in children [4]. The association between health insurance coverage and school absenteeism, however, remains poorly understood. Current research has called for further evaluation of this topic. 

Our objective is to evaluate the association between not having health insurance coverage or having gaps in health insurance coverage and an increased number of missed school days in children. We believe that children of low-income households will be more likely to encounter barriers to health care access, which makes them more likely to miss school due to illnesses or injuries. If this association can be established, we may elucidate the importance of closing the academic gap via efforts that expand health care coverage in school-aged children.

## Materials and methods

A historical cohort study was done via secondary analysis of data collected by the National Institutes of Health (NIH) National Center for Health Statistics. We sampled children enrolled in school, who were between the ages of 6-17 years and who provided answers collected by the NIH National Center for Health Statistics, as part of the 2018 National Survey of Children’s Health (NSCH) involving our two variables of interest: health insurance status and missed school days. Our research qualified for IRB exemption because we used secondary analysis of data and did not directly study human participants. 

The data were collected in the 2018 NSCH, which collects data on children of ages 0-17 years in the United States. A screener questionnaire, as part of the NSCH, was distributed to households throughout the United States initially in order to determine which residents have children aged 0-17 living at the address. The surveys distributed were age-specific and were divided into three age ranges: 0-5 years; 6-11 years; and 12-17 years. Information about the children’s demographics was obtained from the Census Master Address File that covered all 50 states in the United States and the District of Columbia, which was originally obtained from knowledgeable parents or guardians. The sample consisted of 176,000 households, which was stratified by a “child-presence indicator” in order to increase the number of households with children in the sample. The NSCH surveyed the parents, and a list of children in the household was obtained with one child who was randomly selected to be part of the data set. The surveys were available in both English and Spanish.

We excluded children who were not enrolled in school - children aged 5 and under - and any participants whose parent or legal guardian did not provide answers to questions regarding either health insurance status or missed school days. Additionally, we excluded participants who had missing information on health insurance status and missing information on missed school days. Afterward, there were a total of 21,498 children who met our inclusion and exclusion criteria. 

The independent variable in our study is health insurance status as reported in the survey. The following question was used to obtain the data: “DURING THE PAST 12 MONTHS, was this child EVER covered by ANY kind of health insurance or health coverage plan?” The following answer choices were given: “Yes this child was covered all 12 months,” “Yes, but this child had a gap in coverage,” and “No”. The exposure group included those who either did not have health insurance coverage or had gaps in their coverage throughout the 12 months of the data collection. The control group included those who had consistent health insurance coverage throughout the study.

The dependent variable was missed school days. The following question was used to obtain the data: “DURING THE PAST 12 MONTHS, about how many days did this child miss school because of illness or injury? Include missed days from any formal homeschooling.” The following answer choices were given: “No missed school days,” “1-3 days,” “4-6 days,” “7-10 days,” “11 or more days,” and “This child was not enrolled in school”. We used 11 or more missed school days to define and measure our dependent variable. 

Confounding variables that we assessed for included the following: age in years; sex; race; Hispanic ethnicity parental education; adverse childhood events (ACEs); teeth description; food situation in the household within the prior 12 months; use of free/reduced cost meals at school; health-affected ability; general health rated by the parent; family meals eaten together; and special health care need status. 

We used a descriptive analysis to list the baseline characteristics of the population, such as age and sex. We used percentages to describe qualitative variables and calculate means and standard deviations (for variables that are normally distributed) and medians and ranges (for variables that are not normally distributed) to describe quantitative variables.

A bivariate analysis was used to determine the association between baseline characteristics/confounding variables and exposures (lack of or gaps in health insurance coverage). We also examined the association between the exposure or baseline characteristics / confounders and the outcome (missed school days). We defined increased missed days of school as missing greater than or equal to 11 days of school within the past academic year. Statistical significance for confounding variables was considered for p<0.1.

A multivariable regression analysis, using logistic regression, was used to determine and model existing relationships between confounding variables and the association between the exposure and the outcome. Determining this relationship and measuring its effect demonstrated any existing association between lack of or gaps in health insurance coverage and missed school days and avoided overestimation. Statistical significance was considered for p<0.05. 

## Results

An initial sample size of 21,954 respondents was considered. Of these, 456 were excluded due to not being enrolled in school, missing information on insurance status, and missing information on missed school days. A total of 21,498 respondents were included in our final sample. 

The data in Table 1 compare the baseline characteristics according to health insurance status. In summary, the proportion of Blacks and non-White was highest among those who had gaps in insurance or no insurance throughout the entire year. When analyzing the influence of parental education on the insurance status of school-aged children, it was found that children of parents with a lower degree of education were more common among the health insurance gap/uninsured group. In contrast, the frequency of children with special health needs was higher in the group with health insurance. There was no significant difference between those with insurance compared to those without insurance or with gaps in insurance over the past year for the following variables: age, sex, ACEs, health-affected abilities, or general health. Of note, the ACE total consisted of a scale that combined all of the ACE variables of interest together. 

**Table 1:** Baseline characteristics of US primary and secondary school students (ages 6-17) who are insured or uninsured with or without gaps in insurance status (N= 21,498) SD: Standard deviation

Characteristics	Value	Insurance Status					p-value
		Insured			Gaps/No insurance		
		N		%	N	%	
Demographics							
Age - years, mean (SD)		11.5 (3.5)			11.7 (2.7)		0.469
Sex	Male	10,570		50.9	699	51.3	0.878
Race	White	15,742		67.7	903	57.1	0.001
	Black	1,340		13.7	156	19.3	
	Other	3,091		18.6	266	23.6	
Hispanic		2,268		24.5	265	41.5	<0.001
Parental education	Less than high school	494		9.4	129	24.6	<0.001
	High school	2,669		18.0	336	29.2	
	Some college or associate degree	4,859		22.6	402	20.2	
	College degree or higher	12,151		29.9	458	25.9	
Adverse childhood events (ACEs)							
Hard to cover basics like food or housing	Never	10,733		50.9	465	39.4	0.001
	Rarely	6,666		33.4	502	41.0	
	Often	2,519		15.7	330	19.6	
Treated unfairly because of race		830		5.1	73	5.6	0.665
Parent or guardian divorced		5,497		29.4	450	33.8	0.139
Parent or guardian died		753		3.9	71	5.1	0.179
Parent or guardian time in jail		1,494		9.1	178	11.8	0.052
Child experienced ‐ adults slap, hit, kick, punch others		1,162		6.9	135	8.5	0.192
Victim of violence		879		5.3	115	8.1	0.029
Lived with mentally ill		2,015		9.1	160	10.0	0.558
Lived with a person with an alcohol/drug problem		2,257		10.1	204	10.2	0.956
ACE Total	0	7,404		36.8	295	26.9	0.011
	1	6,134		32.0	421	37.6	
	2 or more	5,648		31.2	514	35.5	
Health and safety of child							
Teeth description	Excellent / very good	16,344		76.8	901	75.1	<0.001
	Good	2,952		17.4	274	21.0	
	Fair / poor	825		5.9	147	13.6	
Health-affected ability	This child does not have any conditions		13,043	67.3	864	68.1	0.798
	Never		3,333	16.4	207	16.4	
	Sometimes		2,858	12.4	177	10.9	
	Usually/Always		739	3.9	54	4.7	
Special health care needs status			5,550	23.5	312	17.0	0.001
General health	Excellent		18,353	89.6	1167	86.7	0.134
	Good		1,490	8.8	123	10.8	
	Fair/Poor		269	1.6	33	2.5	
Food and meals							
Family meals eaten together	0 days		729	3.8	61	3.9	0.035
	1-3 days		5,578	26.5	335	25.4	
	4-6 days		7,037	31.1	393	24.4	
	Everyday		6,605	38.6	508	46.3	
Free / reduced cost meals			4,600	35.5	510	46.9	<0.001
Food Situation in the Household (past 12 m)	Could always afford to eat good nutritious meals		14,656	67.4	718	57.6	0.001
	Could always afford enough to eat but not always the kinds of food we should eat		4,560	27.6	470	36.7	
	Sometimes could not afford enough to eat / Often we could not afford enough to eat		703	5.0	101	5.8	

Table 2 shows the association of insurance status and other baseline characteristics with school absenteeism among the samples over an entire year. Although not statistically significant, the percentage of children with greater than or equal to 11 missed days of school was higher in those that were uninsured (5.1%) than in children with insurance (4.4%). The average age of children with chronic absenteeism was significantly higher compared to that of children without chronic absenteeism. Children whose parents had a high school diploma or some college as their highest academic achievement had more absenteeism compared with children whose parents had less education, no high school degree, or more education (such as a bachelor's degree or higher). There was a higher prevalence of chronic absenteeism in children who experienced any form of ACEs, as well as among those with two or more ACEs, compared to those who experienced less than or equal to one ACE. Children from households with difficulties regarding food or that were in free or reduced-cost meal programs also experienced higher frequencies of absenteeism. Finally, children with a fair/poor description of their teeth and who have health-affected abilities had a higher prevalence of chronic absenteeism. There was no significant difference in the frequency of chronic absenteeism by age, race, sex, Hispanic ethnicity, general health, and family meals eaten together. 

**Table 2:** Baseline characteristics of US primary and secondary school students (ages 6-17) who missed less than or greater than 11 school days in a 12-month period (N= 21,498) *Statistically associated with both the exposure and outcome (potential confounder) SD: Standard deviation

Characteristics	Value		Missed days of school in a 12-month period												
			≤11 days missed						>11 days missed						p-value
			N			%			N			%			
Insurance Status	Insured		19,255			95.6			918			4.4			0.511
	Gaps/No insurance		1,250			94.9			75			5.1			
Demographics															
Age - years, mean (SD)			11.5 (3.4)						12.9 (3.4)						<0.001
Sex	Male		10,765			95.5			504			4.5			0.856
	Female		9,740			95.4			489			4.6			
Race	White		15,856			95.6			789			4.4			0.545
	Black		1,431			95.9			65			4.1			
	Other		3,218			94.7			139			5.3			
Hispanic	Hispanic		2,417			95.0			116			5.0			0.481
	Non-Hispanic		18,088			95.7			877			4.3			
Parental education	Less than high school		585			96.7			38			3.3			<0.001*
	High school (including vocations, trade, or business school)		2,837			93.6			168			6.4			
	Some college or associate's degree		4,932			94.0			329			6.0			
	College degree or higher		12,151			96.7			458			3.3			
Adverse childhood events (ACEs)															
Hard to cover basics like food or housing	Never		10,923			98.0			275			2.0			<0.001*
	Rarely		6,796			95.1			372			4.9			
	Often		2,519			88.8			330			11.2			
Treated unfairly because of race			835			92.4			68			7.6			0.021
Parent or guardian divorced			5,546			92.6			401			7.4			<0.001
Parent or guardian died			750			91.4			74			8.6			0.001
Parent or guardian time in jail			1,529			90.2			143			9.8			<0.001*
Child experienced ‐ adults slap, hit, kick, punch others			1,161			88.9			136			11.1			<0.001
Victim of violence			867			85.3			127			14.7			<0.001*
Lived with mentally ill			1,926			87.1			249			12.9			<0.001
Lived with a person with an alcohol/drug problem			2,256			92.0			205			8.0			<0.001
ACE Total	0			7,545			98.6			154			1.4		<0.001*
	1			6,276			95.9			279			4.1		
	2 or more			5,662			91.7			500			8.3		
Health and safety of child															
Teeth description	Excellent/Very Good			16,617			97.0			628			3.0		<0.001*
	Good			2,982			93.1			244			6.9		
	Fair/Poor			855			85.0			117			15.0		
Health-affected ability	This child does not have any conditions			13,678			98.3			229			1.7		<0.001
	Never			3,411			96.0			129			4.0		
	Sometimes			2,628			86.1			407			13.9		
	Usually/Always			568			72.2			225			27.8		
Special health care needs status				5,184			87.4			678			12.6		<0.001*
General health	Excellent			18,913			97.3			607			2.7		<0.001
	Good			1,372			85.9			241			14.1		
	Fair/Poor			161			53.7			141			46.3		
Food and meals															
Family meals eaten together	0 days			706			88.9			84			11.1		<0.001*
	1-3 days			5,598			94.8			316			5.2		
	4-6 days			7,132			95.8			298			4.2		
	Everyday			6,831			96.4			282			3.6		
Free or reduced cost meals				4,751			93.6			359			6.4		<0.001*
Food situation in the household (past 12 months)	We could always afford to eat good nutritious meals.			14,885			97.0			489			3.0		<0.001*
	We could always afford enough to eat but not always the kinds of food we should eat			4,654			93.6			376			6.4		
	Sometimes we could not afford enough to eat/Often we could not afford enough to eat			694			86.9			110			13.1		

Table 3 depicts the crude and adjusted odds ratios of chronic absenteeism in those who have health insurance compared to those who do not have it or have experienced gaps in insurance coverage throughout the year. The unadjusted odds of chronic absenteeism were found to be higher in children without insurance or with gaps in insurance compared to children with consistent insurance throughout the year, but the association was not statistically significant. After adjustment for confounders and other variables included for face validity, the odds of chronic absenteeism in children without insurance or with gaps in insurance remained statistically insignificant. Incidentally, our study found that other factors contribute to school absenteeism including two or more ACEs, special health care needs status, health-affected ability, and compromised general health. The unadjusted odds of chronic absenteeism were found to be 16% (OR=1.16) higher in children without insurance or with gaps in insurance compared to children with consistent insurance throughout the year, but the association was not statistically significant (95% CI 0.74 - 1.82, p=0.051). After adjustment by age, sex, race, Hispanic ethnicity, parental education level, and various ACEs, the odds of chronic absenteeism in children without insurance or with gaps in insurance remained statistically insignificant (aOR=1.05; 95% CI 0.64 - 1.73, p=0.848) compared to those with consistent insurance coverage.

**Table 3:** Unadjusted and adjusted associations between school absenteeism and baseline characteristics SD: Standard deviation

Characteristics	Value	Unadjusted		Adjusted	
		OR (95% CI)	p-value	OR (95% CI)	p-value
Insurance status	Insured	reference		reference	
	Gaps/No insurance	1.16 (0.74-1.82)	0.511	1.05 (0.64-1.73)	0.848
Demographics					
Age - years, mean (SD)		1.14 (1.09-1.19)	<0.001	1.10 (1.05-1.15)	<0.001
Sex	Male	reference		reference	
	Female	1.03 (0.77-1.37)	0.855	NA	NA
Race	White	reference		reference	
	Black	0.92 (0.61-1.39)	0.696	0.58 (0.35-0.96)	0.034
	Other	1.21 (0.77-1.92)	0.409	1.04 (0.73-1.49)	0.813
Hispanic		1.15 (.77-1.72)	0.482	1.04 (0.70-1.54)	0.832
Parental education	Less than high school	0.98 (0.58-1.67)	0.947	0.61 (0.32-1.16)	0.134
	High school (including vocations, trade, or business school)	2.01 (1.37-2.93)	<0.001	1.45 (0.96-2.19)	0.075
	Some college or associate's degree	1.87 (1.32-2.65)	<0.001	1.27 (0.92-1.75)	0.146
	College degree or higher	reference		reference	
Adverse childhood events (ACEs)					
Hard to cover basics like food or housing	Never	reference		reference	
	Rarely	2.57 (1.87-3.53)	<0.001	1.39 (0.89-2.18)	0.148
	Often	6.29 (4.43-8.93)	<0.001	1.63 (0.96-2.75)	0.07
Treated unfairly because of race		1.82 (1.09-3.05)	0.023	0.99 (0.61-1.59)	0.956
Parent or guardian divorced		2.33 (1.74-3.11)	<0.001	NA	NA
Parent or guardian died		2.07 (1.32-3.26)	0.002	NA	NA
Parent or guardian time in jail		2.66 (1.67-4.25)	<0.001	0.99 (0.61-1.59)	0.956
Child experienced ‐ adults slap, hit, kick, punch others		2.98 (1.82-4.88)	<0.001	NA	NA
Victim of violence		4.27 (2.55-7.13)	<0.001	1.16 (0.73-1.84)	0.534
Lived with mentally ill		4.01 (2.90-5.55)	<0.001	NA	NA
Lived with a person with an alcohol/drug problem		2.02 (1.44-2.83)	<0.001	NA	NA
ACE Total	0	reference		reference	
	1	2.93 (2.05-4.18)	<0.001	1.38 (0.87-2.20)	0.175
	2 or more	6.25 (4.45-8.71)	<0.001	1.68 (0.94-3.00)	0.079
Health and safety of child					
Teeth description	Excellent/Very Good	reference		reference	
	Good	2.37 (1.74-3.22)	<0.001	1.12 (0.77-1.62)	0.562
	Fair/Poor	5.63 (3.50-9.06)	<0.001	1.95 (1.18-3.23)	0.009
Health-affected ability	This child does not have any conditions	reference		reference	
	Never	2.45 (1.46-4.09)	0.001	1.80 (1.12-2.89)	0.016
	Sometimes	9.55 (6.93-13.17)	<0.001	4.17 (2.76-6.29)	<0.001
	Usually/Always	22.9 (14.40-36.50)	<0.001	6.72 (4.01-11.27)	<0.001
Special health care needs status	Yes	6.73 (5.03-9.01)	<0.001	1.60 (1.10-2.35)	0.016
General health	Excellent	reference		reference	
	Good	5.84 (4.22-8.08)	<0.001	1.9 (1.29-2.2.68)	0.001
	Fair/Poor	30.6 (17.31-54.01)	<0.001	5.42 (3.00-9.78)	<0.001
	Fair/Poor	30.6 (17.31-54.01)	<0.001	5.42 (3.00-9.78)	<0.001
Food and meals					
Family meals eaten together	0 days	3.39 (1.92-5.99)	<0.001	1.91 (1.04-3.48)	0.035
	1-3 days	1.50 (1.04-2.15)	0.031	1.19 (0.84-1.69)	0.325
	4-6 days	1.18 (0.80-1.74)	0.404	1.09 (0.76-1.57)	0.632
	Everyday	reference		reference	
Free or reduced cost meals	Yes	1.94 (1.46-2.58)	<0.001	0.93 (0.67-1.29)	0.66
Food situation in the household (past 12 months)	We could always afford to eat good nutritious meals.	reference		reference	
	We could always afford enough to eat but not always the kinds of food we should eat	2.21 (1.67-2.94)	<0.001	1.10 (0.77-1.58)	0.592
	Sometimes we could not afford enough to eat/Often we could not afford enough to eat	4.92 (2.73-8.85)	<0.001	1.21 (0.69-2.13)	0.513

## Discussion

We suspected that there was an association between not having health insurance coverage and school absenteeism in children. However, our analysis shows that a lack of health insurance or gaps in insurance coverage were not statistically associated with chronic absenteeism. Our findings do not support our hypothesis of a significant difference in missed school days among those children who had health insurance compared to those without health insurance. 

There are no previous studies that specifically investigate the relationship between insurance status and chronic absenteeism in children. However, previous studies surrounding this topic provide insight into our findings. In a study by Olson et al., gaps or lack of insurance coverage was correlated with poor access to adequate health care [5]. Olson found that subjects with poor access to adequate health care were more likely to come from minority groups and low-income households. The relationship described by Olson et al. may contribute to an explanation for the findings in our research. Difficulty accessing health care combined with low-income households may force parents of acutely or chronically ill children to choose between taking a sick child to school or taking the sick child to the doctor. Poor access to health care may favor the decision to send sick children to school. This phenomenon could potentially contribute to minimizing the prevalence of chronic absenteeism in children with gaps in insurance coverage or no insurance coverage at all. 

The study performed by Howell et al. explores this relationship further by examining the resulting effects of an expanded health insurance program for California children. Ultimately, the study showed that improved access to health care led to improved health outcomes and fewer missed school days [6]. Because this study explores the intimate relationship between health status and the effects it can have on school absenteeism, it served as a foundational study for our research objective. Both studies utilized a similar study design; however, our study approach differed significantly from the study performed by Howell et al. Our study analyzed a broad population of children across various socioeconomic statuses in the United States, while the research by Howell et al evaluated children limited to three California counties, focusing on undocumented children and those who belong to a higher income group but still do not qualify for the state’s Medicaid program or Children’s Health Insurance Program. Such differences in study design and statistical outcomes encourage further study on the topic of children’s health care and the potential impact it may have on school absenteeism and therefore, potential social mobility. 

Our study highlights this issue by further supporting the association between chronic school absenteeism and adverse childhood experiences among school-age children that has been elucidated by Stempel et al. [7]. After adjusting for confounders, we may add to the existing evidence that children who have experienced two or more ACEs are more likely to suffer from chronic absenteeism. This finding implies that in order to improve school attendance and promote the social mobility of children, we must address childhood adversities. This will require a multidisciplinary approach that can recognize the psychosocial factors children encounter in their communities, homes, and outside of school grounds. 

Furthermore, we hypothesized that parents with less education were more likely to have limited flexibility in working hours and paid leave, making them more likely to send their children to school regardless of any underlying health issues. On the other hand, parents with a higher education level were more likely to be able to afford alternative childcare options if a child was not well enough to attend school. However, our data did not support this after adjusting for confounding variables. There were, on the other hand, statistically significant differences in chronic absenteeism among children who experienced two or more adverse childhood events, had special health care needs status, health-affected ability, or compromised general health. Given the interesting relationships, these topics would benefit from further research to explore the impact that ACEs have on the educational level that these children achieve and as a result, the likelihood of future social mobility. 

Strengths 

A significant strength of our study is that it is the first to explore the association between health insurance status and missed school days in school-aged children. Prior studies have investigated other factors that contribute to children’s school attendance, including but not limited to race, household income, parental education, overall health status, family structure, adverse childhood events, and dental insurance coverage [7,8]. However, they do not specifically examine whether health insurance status may act as an indicator of a child’s school attendance and performance, which can ultimately negatively impact their future career opportunities and social mobility - a well-established association [9,10]. The results of our study bring awareness and highlight the need for additional studies on this topic. 

The publicly available database collected by the National Institutes of Health (NIH) National Center for Health Statistics makes our study sample similar to the general population of school-aged students in the United States, given the inclusion of all 50 states. In addition, the survey was self-reported and anonymous implicating that families presumably felt comfortable reporting answers. The survey was available in English and Spanish, which expands its reach to families of diverse backgrounds. Additionally, this survey asked a number of questions that address potential confounders to our study hypothesis, which were adjusted for in our analysis. We analyzed confounding variables using a more liberal baseline by using a p-value of 0.1. This allowed us to include and capture all possible confounding variables while reducing the chance of type I error. 

Limitations

The data from our study were obtained from the NSCH. The U.S. Census Bureau now conducts the NSCH annually on behalf of HRSA MCHB and HHS under Title 13. This data source serves as a limitation for our study because it excludes portions of the population. As a result, the frequencies of our independent variable -- lack of health insurance or gaps in insurance coverage -- and our dependent variable -- chronic absenteeism were both small at 6.2% and 4.6%, respectively. The excluded populations likely include undocumented or documented immigrants who may fear legal repercussions for making their status known to a governmental agency.

All households selected to participate in the 2018 NSCH received a mailed invitation to respond to the survey by web. This may have deterred families without access to the internet to complete the survey which is more likely to occur in lower-income households. 

Generalizations

The data analyzed in this study were collected by the NSCH which gathers data on children throughout all 50 states in the United States on an annual basis. This allows us to generalize our results to children across the United States. Our study explored the association between health insurance coverage and school attendance. We were interested in seeing whether this could have policy implications that could warrant the expansion of health insurance coverage among children.

The demographic data suggest that race influences both health insurance status and missed school days. Although the findings were not statistically significant, the impact that race has an impact on health insurance coverage as they may be an avenue for future exploration. Validation of the relationship between race and both insurance status and missed school days can possibly influence changes in health policy to support children of racial minorities, including Blacks and Hispanics those impacted by adverse childhood events, and whose parents are less educated, in order to help facilitate social mobility and lessen the impact of social determinants of health. 

## Conclusions

Despite there being no conclusive evidence to suggest that lack of health insurance leads to more missed school days, our study incidentally highlights other variables that impact children’s school attendance. The data suggest that the presence of ACEs, compromised general health, and special health care needs are implicated in chronic absenteeism, which is consistent with existing literature. 
